# Effectiveness of Dowels in Concrete Pavement

**DOI:** 10.3390/ma12101669

**Published:** 2019-05-22

**Authors:** Jiri Grosek, Andrea Zuzulova, Ilja Brezina

**Affiliations:** 1 Transport Infrastructure Department, Transport Research Centre, 63 600 Brno, Czech Republic; ilja.brezina@cdv.cz; 2 Department of Transportation Engineering, Faculty of Civil Engineering, Slovak University of Technology in Bratislava, 810 05 Bratislava, Slovakia; andreazuzulova@gmail.com

**Keywords:** concrete pavement, dowel, load transfer, finite element method

## Abstract

Dowels are located in transverse joints of Jointed Plain Concrete Pavements (JPCP) and they are used to provide load transfer between individual slabs, reduce faulting and improve performance. Dowels and the concrete itself are under the highest stress in the vicinity of joints; thus, in terms of pavement design, the joints are the weakest points of the whole structure. This study dealt with the drawbacks of JPCP with dowels. The evaluation was based on direct measurements on real airport and motorway pavements and highlights insufficient efficiency of load transfer and its possible causes. The authors present a successful outcome with validation by using the finite element method where high tensile stress values of the surrounding concrete were found.

## 1. Introduction

Jointed plain concrete pavement is typically composed of a Portland Cement Concrete (PCC) surface course constructed on top of either an underlying base course or in exceptional cases on the subgrade. Joints of concrete pavement are stressed by the effects of overruns of heavy vehicles and temperature and moisture gradient of concrete slabs. The dowels in transverse joints must allow horizontal movement of slabs in the contracting time after the pavement is paved and during its further use. In addition, they reduce vertical movements on slab edges and occurrence of vertical unevenness along the pavement lifecycle. The monitored parameter is the transfer of load on joints expressed by the value of LTE (Load transfer efficiency).

The loaded and adjacent unloaded slabs deflect when a wheel load is applied at a joint edge. Deflections of jointed slab are directly coherent to joint performance. When the loaded and unloaded slabs deflect equally, the joints performance is accomplished. Load transfer efficiency may be described by the following equation:

(1) $$\mathrm{LTE}\;\left(\%\right)=\frac{d_{u}}{d_{l}}\times 100$$

where *d_l_* is the loaded slab and *d_u_* is the adjacent unloaded slab deflection [1].

The LTE depends on a number of circumstances, including the base course or subgrade support, aggregate interlock, joint width and spacing, temperature and moisture (which could affect joint opening), number of passes and load intensity, and the methodology of measurement through the load transfer devices (falling weight deflectometer, FWD). Several studies describe the most performance problems with concrete pavement as a result of poor performance of transverse joints [2,3]. A poor LTE may cause higher slab stresses, which may strongly contribute to distresses, such as faulting, pumping, corner breaks and roughness. Thus, LTE requires checking to achieve long-term concrete pavement performance.

Long-term evaluation of the measurement results on tested concrete pavements of airport runways and motorways shows that the LTE values range between 0.70 and 0.90. According to the current Czech technical specification TP 92, efficient transfer should exceed 0.90, otherwise the structures should be evaluated to decide whether a measure is necessary. This means that the majority of joints fail to meet this criterion. The evaluation of the current study is based on direct measurements by falling weight deflectometers (FWD) made by companies KUAB and Rodos [4].

The issue of LTE is dealt with by many authors and organizations [5,6]. The authors of [7,8,9,10,11,12,13] described long-term monitoring of potential alternative dowels to be used in concrete pavements to increase load transfer efficiency and to monitor resistance of dowels against defrosting agents mainly related to steel dowels. However, long-term corrosion protection is provided by epoxy coatings along the whole length of the dowel. A slab failure due to dowel corrosion has not been confirmed in the Czech Republic.

In addition, the results show that in many cases LTE is low when different types of dowels are used. The reasons include the behavior of dowels at overruns of heavy vehicles, the quality of subbase and subgrade, and the quality of dowels, their coating and alignment. In Slovakia, recommended dimensions of dowels (diameter and length) are applied to concrete slab thickness; in the Czech Republic, a unified diameter (25 mm) and length (500 mm) of dowels are used for all pavement thicknesses [14,15,16,17,18].

Some studies and recommendations also suggest that the position of dowels towards the transverse joint significantly affects the performance. The alignment of dowel bars is important since significant misalignment of dowels in a doweled joint may prevent that joint from opening/closing properly. The occurrence of a single joint that does not open/close effectively will not necessarily result in a mid-panel crack or another pavement defect, but the risk of mid-panel cracking and joint distress increases with each successive joint with limited opening/closing capabilities. The vertical tilt is particularly critical, as well as the vertical and longitudinal translation of the dowels [19].

Software based on the principle of the finite element method (FEM), such as SCIA Nexis, ANSYS, etc., allow modeling of stress in concrete pavements, which can be used for making comparisons of calculated and real values. The FEM calculation allows monitoring stress conditions of concrete in terms of load and to compare different types of structures and used dowels (dimensions, coating, placement, etc.). 3D FEM is an efficient and accurate method to calculate the interaction characteristics in concrete pavements. Research activities tend to study tensile stress concentration of the surrounding concrete around dowels and potential to cracking. A small diameter, high strength of dowels and their position are considered the main reasons for failure [20,21,22,23,24,25,26,27].

The aim of the research activities of the authors from the Czech Republic and Slovakia was to carry out measurements on test sections, present the experience with modeling in software based on the principle of FEM and inform of new findings regarding dowels. In addition, we aimed to point out and investigate the differences in tensile stress on the bottom part of concrete slabs and around dowels with defectively installed dowels, which are crucial for design [28,29].

## 2. Materials and Methods

### 2.1. LTE Measurement on Existing Pavements Methodology

The aim of the measurements was to evaluate bearing capacity and determine LTE of concrete slabs on airport runways (RWY), taxiways (TWY) and aprons in Slovakia, and road sections complying with motorway design standards in the Czech Republic.

#### 2.1.1. Bratislava Airport

An example of a “heavy loaded” area of concrete pavement includes an airport runway. Deformations measured by dynamic load on the slab edge (at the joint) and recorded on the adjacent slab depend mainly on the construction design of joints. The transverse joints were reinforced with dowels while the longitudinal joints were without any reinforcement on a RWY section selected for experimental measurements.

**Figure 1:**
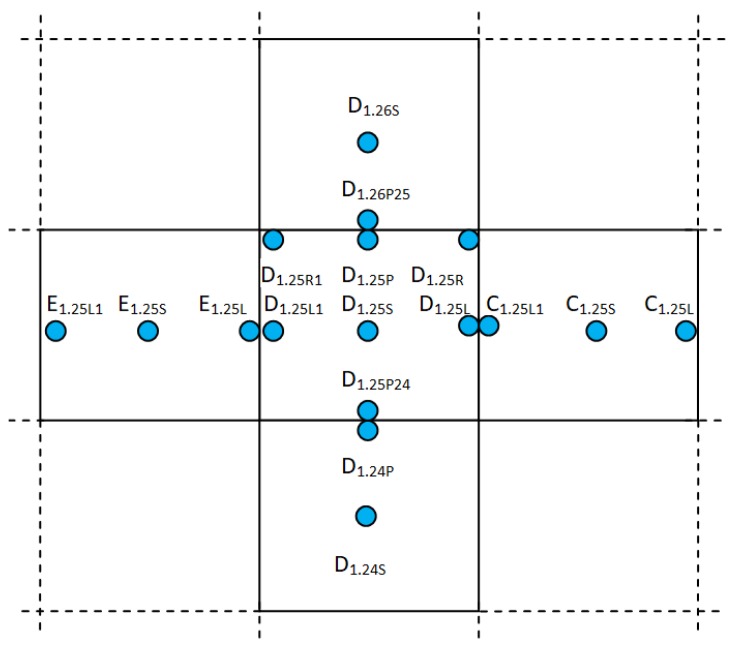
Locations of falling weight deflectometer (FWD) load plate on runway (RWY).

Dynamic load tests with the aim to measure LTE between slabs were carried out by FWD KUAB (KUAB Konsult & Utveckling AB, Rättvik, Sweden) with the average load force of 150 kN and with the load duration of 60 ms. According to the differences in the pavement structure—mainly in the slab thickness (220, 240 and 300 mm)—the runways were divided into sections. A configuration scheme of five tested slabs is shown in Figure 1.

**Figure 2:**
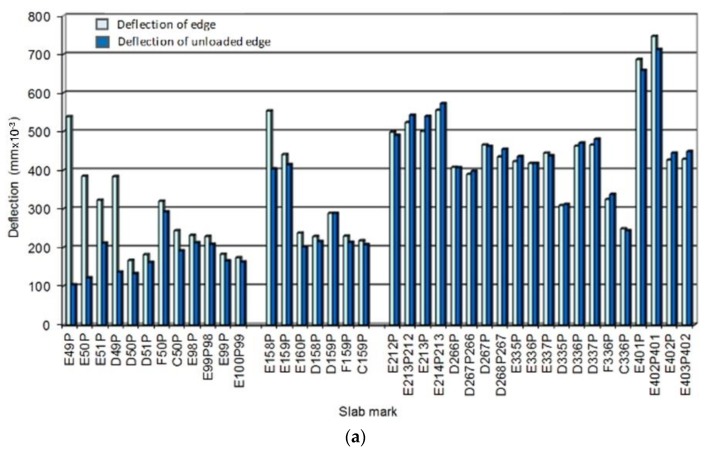

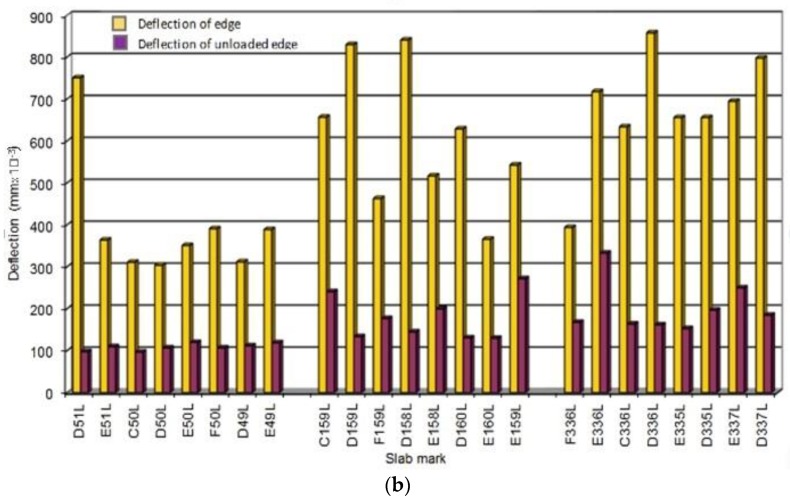
(**a**) Load transfer across transverse joints of RWY 13-31; and (**b**) load transfer across longitudinal joints (without any reinforcement) for RWY 13-31

LTE on transverse joints was higher than 80% and, on some paths of RWY (JPCP thickness 300 mm), it was higher than 92% (Figure 2a). LTE on longitudinal joints was low, on RWY only 32% on average (Figure 2b). This should be taken into consideration in the reconstruction design of this runway.

#### 2.1.2. Highway D1 and R1

**Figure 3:**
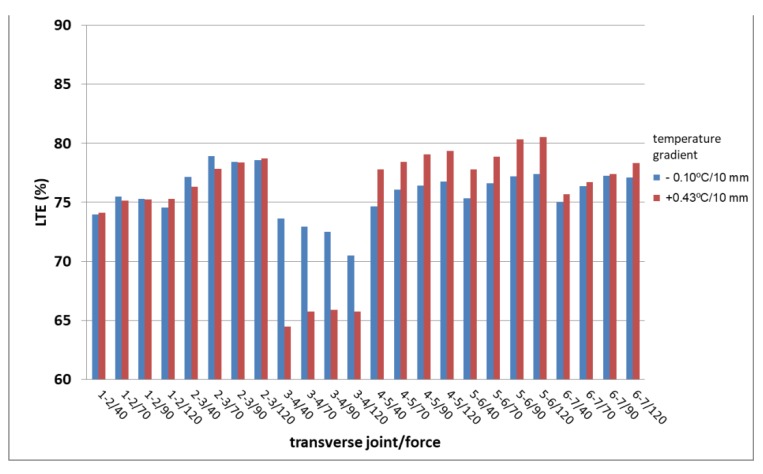
Evaluation of LTE on Highway D1—comparisons of different load and temperature gradients.

The evaluation of LTE on edges of six slabs with the FWD/HWD RODOS 10001 (Ing. Pavel Herrmann—RODOS, Prague, Czech Republic) was performed. Low values of load transfer on transverse joints reinforced with dowels (slab thickness 250 mm) were found on two sections on R1 and D1. The effect of different load (40, 70, 90 and 120 kN) and temperature (gradient −0.10 and +0.43 °C/10 mm) were also studied (Figure 3). The measurements used a load plate of 300 mm diameter and the evaluation of LTE was considered as a ratio of deflections on geophones in distances of 300 and 200 mm from the center of load, while the transverse joint is located in between these geophones (transverse joint at the distance of 250 mm from the load point). The results show low values and the reason is difficult to find. Nevertheless, it is probably a combination of several unfavorable factors which influence LTE value, such as effect of quality of subbase and subgrade on dowels.

The measurement results from the test sections can be used for modeling in software based on the finite element method and can be focused on specific and problematic parts of the structure.

### 2.2. Modeling of Stress on Slab Edges by FEM Methodology

The modeling included an analysis of stress on concrete slab edges while using findings from field measurements on the test sections. The software allows modeling of stress at the bottom part of slabs by traffic and temperature load and can account for the interaction of pavement and subgrade.

#### 2.2.1. Software SCIA Nexis

Different degrees of interaction of concrete slabs were considered for the selected pavement structure. The result of such modeling is a numeric and graphic representation of concrete slabs behavior (stress and deflections).

The calculation was made by FEM method with the use of SCIA Nexis software. The modeling of the interaction of concrete slabs and subbase is reduced to a 2D model, whose special case is the Pasternak model. Pasternak’s characteristics of subbase properties with two parameters (C1 and C2) describe the behavior of slabs better, particularly on solid subbase. The authors of the software, Kolar and Nemec, produced a multi-parameter 2D model, which is equivalent to half-space. Selecting proper constants of C1 and C2, it is possible to draw close to the real deformation of slabs.

The constant C parameters influence the stress and the distribution stress has an effect on the settlement of the base (subbase). Therefore, an iterative equation is used for the constant C. For the calculation module Soilin and the relationship between the constants of the model (3D) and surface model (2D) the following equations were used:

(2) $$C_{1z}^{S}=\overset{H}{\underset{0}{\int }}E_{z}{\left(\frac{\partial f\left(z\right)}{\partial z}\right)}^{2}dz$$

(3) $$C_{2x}^{S}=C_{2y}^{S}=\overset{H}{\underset{0}{\int }}Gf^{2}\left(z\right)dz$$

where *E_z_* is the deformation modulus of soil, *G* is the shear modulus, and *H* is the depth of deformed zones of the spatial model “damping function” *f*(*z*), which is defined by the slump ratio at a given depth *w*(*x*,*y*,*z*) for settlement surface *w*_0_(*x*,*y*) in the form:

(4) $$f\left(z\right)=\frac{w\left(x,y,z\right)}{w_{0}\left(x,y\right)}$$

Unlimited slide is considered between the slabs and the subbase, which helps to get the most unfavorable stress values in the slab. This phenomenon usually occurs in practice, particularly regarding the dilation of slabs and warping of slabs due to temperature gradient.

Another step is the modeling of transverse and longitudinal joints which break the integrity of the pavement. The selected 2D macro can be assigned with a constant thickness (constant isotropy), variable thickness (variable isotropy) and orthotropy. In the cases when the constant isotropy is assigned, 2D macros will be assigned with a constant thickness and material (quality). In the case orthotropy is considered, macros will be assigned with thickness parameters using the values of stiffness matrix elements. This is method allows modeling any joint.

#### 2.2.2. Software ANSYS

**Figure 4:**
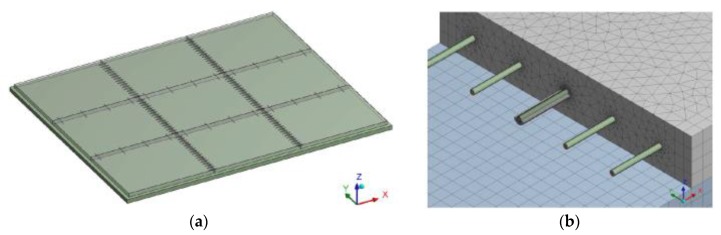
(**a**) Global; and (**b**) detailed numeric model.

The calculation included an analysis of static stress at the bottom part of the concrete slab while considering defective positions of dowels. Software ANSYS, with the use of detail spatial calculation models, modeled pavement structure, which consisted of a mechanically compacted aggregate layer with the thickness of 250 mm and concrete slabs fitted with dowels and tie bars. Tie bars are made of construction steel of 20 mm diameter and 800 mm length, and their central part is covered with a plastic coating. Steel dowels, 25 mm diameter and 500 mm length, are smooth on the surface. The sliding of dowels is allowed by the plastic coating of an average width of 0.4 mm. The global calculation model covers an area formed by 3.0 m × 3.0 m slabs. The detailed, more accurate, model includes a transverse joint with five dowels. The global calculation model includes boundary conditions for the detailed model, global displacement and stress of the area. The cross cut of the concrete slab at the place of transverse and longitudinal joints is considered based on the assumption that at these weaker spots the slabs will be split into separate rectangular segments of 5.5 m × 4.5 m. The thickness of concrete slabs is 240 mm, which is a similar value as the standard used thickness of concrete slabs in the Czech Republic (Table 1). The dowels and tie bars are vertically placed so that the upper part of a steel dowel aligns with the central axis of the slab. The horizontal placement is symmetrical around the joint, whose parameters are shown in Figure 4. Seventeen dowels in total are placed in this way (spacing 250 mm) for a single transverse joint and four tie bars (spacing 1500 mm) for a single longitudinal joint of the segment.

**Figure 5:**
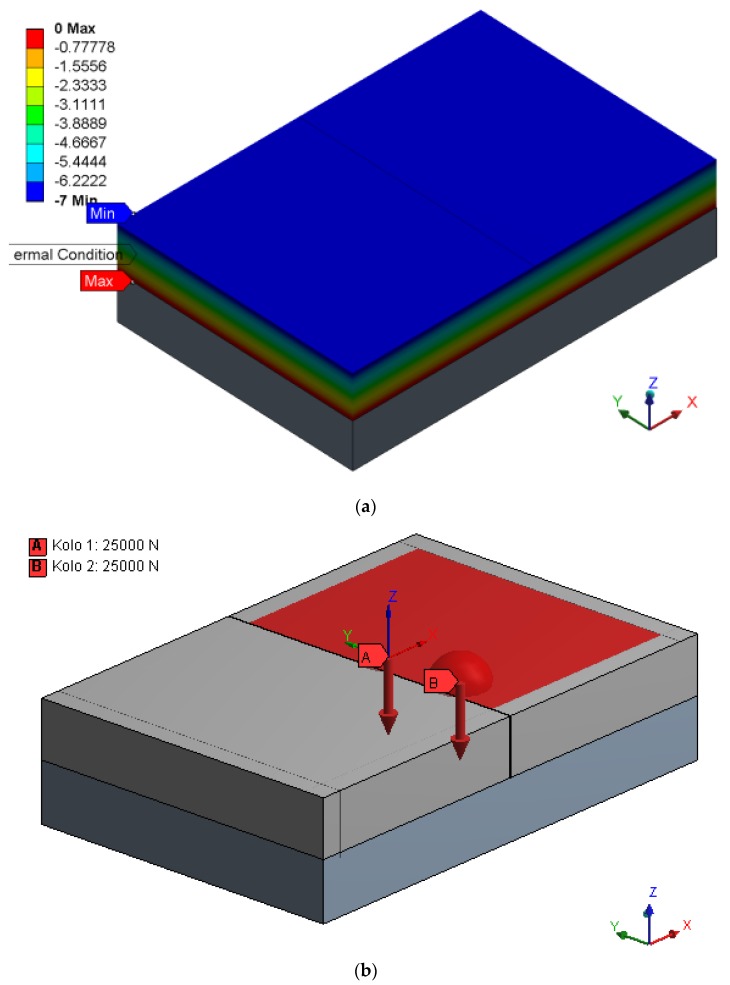
(**a**) Temperature gradient; and (**b**) dual wheel axle on concrete pavement.

The calculation used a boundary condition of the fixed placement in the horizontal direction and flexible placement (Winkler’s model) in the vertical direction. This boundary condition was applied to the bottom part of the subbase aggregate layer. Winkler’s model of subgrade works with modeling of subgrade soil. Linear changes in temperature along the concrete slab thickness were assigned in such way that the temperature of the bottom was 0 °C and the surface −7 °C. The external load by dual wheel axle of 50 kN force, representing load by the standard design axle (Czech design axle), was entered into the numeric model apart from its own weight and temperature load. The axis of one wheel axle intersects the axis of a dowel. The axis of the second wheel axle is located 344 mm apart in the Y direction. The diameter of the contact areas is 240 mm. These areas are located right next to the transverse joint edge of the monitored slab (Figure 5).

Spatial models of the pavement allow performing different variants of solutions with the inclusion of dowels in different positions. Non-linear calculations were performed for 17 variants. A certain variant models the precise position of a dowel according to the project design. Another variant models the missing dowel. Six variants model different vertical translations of dowels. Another six variants model different vertical tilts of dowels. Three variants model different longitudinal translations of dowels. Each of the variants of defective dowels was described by a calculation:

- Global numeric model ZS1 (pavement weight, temperature field);
- Global numeric model ZS2 (load by axle);
- Local numeric model ZS1 (pavement weight, temperature field); and
- Local numeric model ZS2 (load by axle).

**Figure 6:**
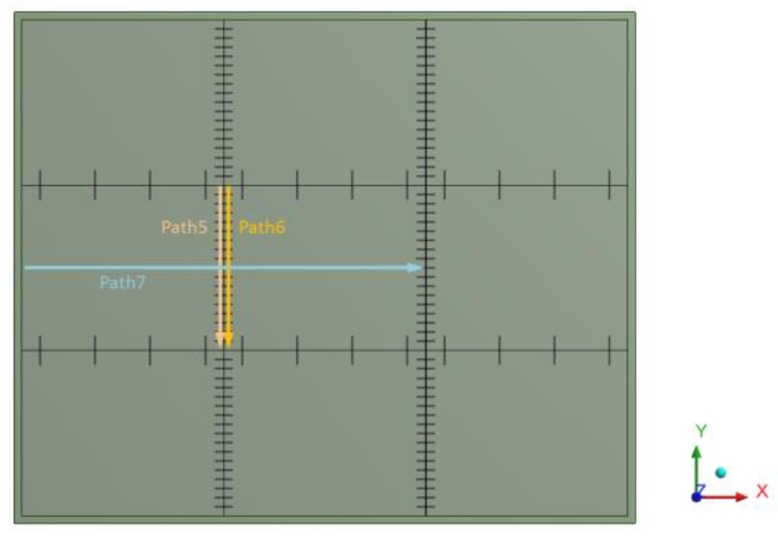
Global positions of paths at bottom parts of concrete slabs.

The goal of the calculation was to observe whether a defective dowel position has an impact on the stress at the bottom part of concrete slab and cracking potential of concrete in the vicinity of the dowel. Figure 6 shows global positions of paths on which the resulting stress at the bottom part of the pavement was assessed.

**Figure 7:**
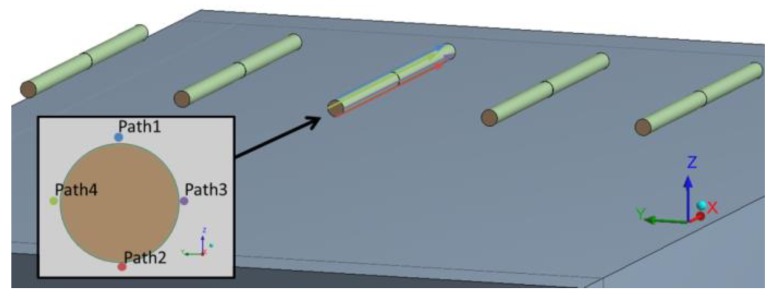
Position of paths led in concrete slab near defective dowel.

An analysis of stress of the concrete around dowels was performed as well. Figure 7 shows the position of paths which were evaluated for stress. The paths were in the close vicinity of default dowels. The paths were always in the direction of the dowel. The path always started at the edge of the dowel on a non-loaded concrete slab. The end of the path was always located at the distance of 500 mm from the front, i.e., at the other edge of the dowel, which was on another concrete slab loaded by dual wheel axle. Four paths were applied: two to get compressive stress and two to get tensile stress.

## 3. Results and Discussion

### 3.1. SCIA Nexis Results

**Figure 8:**
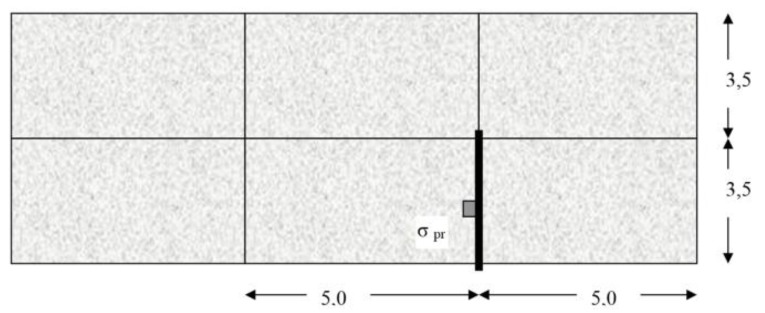
Scheme of concrete slabs.

The mentioned assumptions were applied for the calculation of a set of six slabs. The pavement structure consisted of a concrete layer of 250 mm thickness, laid on a layer of asphalt concrete AC of 50 mm thickness, a cement bound granular mixture CBGM of 180 mm thickness, and a protection gravel course of 270 mm thickness. The scheme of the set of concrete slabs is shown in Figure 8, which also shows the load and the evaluated joint.

**Figure 9:**
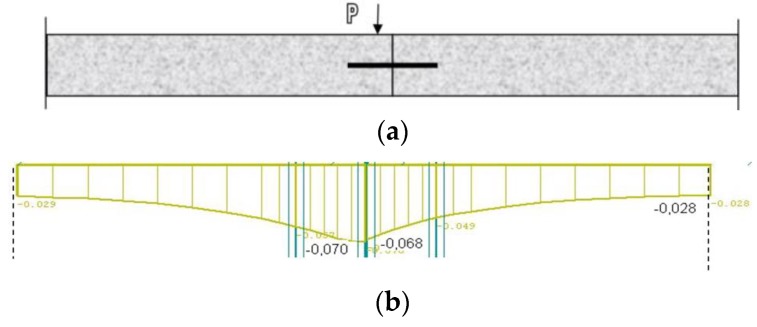

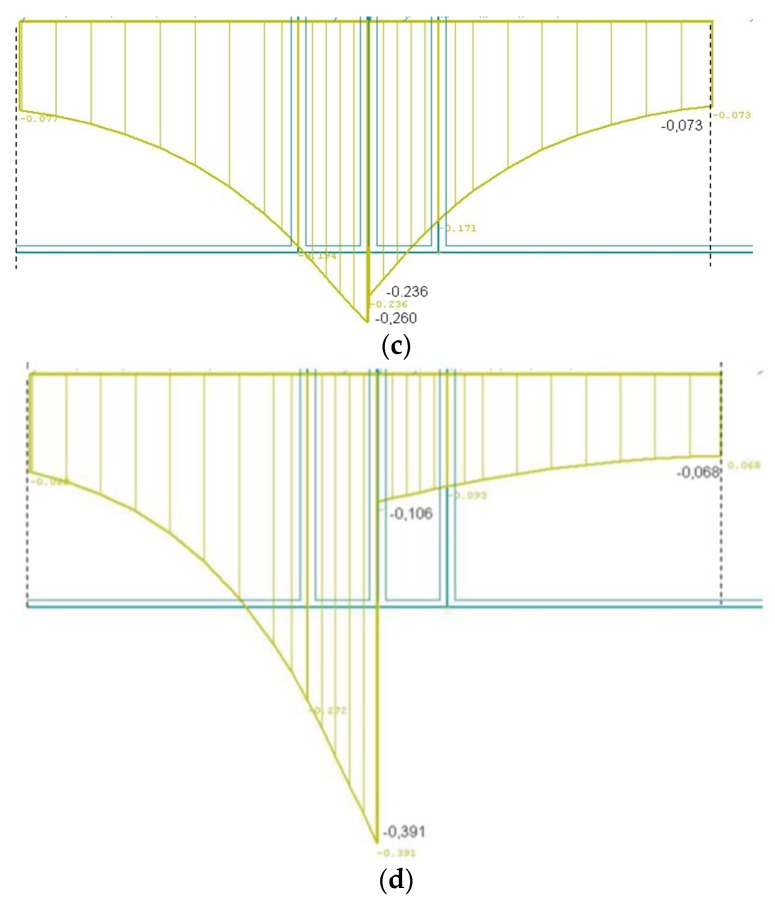
Comparison of deflection on transversal joint of concrete pavement at different degrees of interaction: (**a**) load diagram of transverse joint; (**b**) stiff subbase/good interaction; (**c**) soft subbase/good interaction and (**d**) soft subbase/poor interaction.

The calculations of LTE show the impact of load transfer values on the evaluated parameters. Figure 9 shows a comparison of deflections on the evaluated transverse joint at different degrees of interaction.

In the first case, orthotropy was considered for the evaluated transverse joint without reduced stiffness matrix elements (Figure 9a). In this case, stress had a value of 0.630 MPa on the loaded part of the slab and 0.329 MPa on the adjacent slab, which was around 50% of the value on the loaded slab part. The deflection here reached the value of 0.070 mm on the loaded part and 0.068 mm on the non-loaded slab part, therefore the interaction can be regarded as very good (97%).

In another case, when orthotropy was considered for the evaluated transverse joint, but with reduced stiffness matrix elements, the values of parameters of subbase C1 and C2 near the evaluated transverse joint were reduced as well (Figure 9b). The results show that stress on the loaded slab part increased to the value of 1.157 MPa, but only to the value of 0.429 MPa on the non-loaded part, which was only about 35%. Even though the deflection on this pavement structure was higher in comparison with the previous example, reaching 0.260 mm on the loaded slab part and 0.236 mm on the non-loaded part, it was possible to regard the interaction as very good (90%).

In addition, a situation was modeled when the parameters of the transverse joint were reduced in greater degree and some of the parameters had zero value. As in the previous case, the values of parameters of subbase C1 and C2 near the evaluated transverse joint were reduced, the values equaled zero under the joint (Figure 9c). The calculated stress on the loaded slab part had a value of 1.597 MPa and zero on the non-loaded part. No stress condition occurred on the adjacent slab due to this load. The stress in this case was nearly 1.5 times higher than the stress in the previous case when the structure behaves as a whole unit. The deflection reached 0.391 mm on the loaded slab part and 0.106 mm on the non-loaded part, which was up to four times higher than in the first case. Therefore, the interaction was really poor (27%).

The adverse effect of temperature gradient on slab stress can be reduced [28]. This can occur when designing concrete pavement for inside conditions, e.g., a tunnel. Stress was caused by an axle load of 100 and 115 kN and the temperature gradient inside was 10 °C − 2.2 °C = 7.8 °C (marked as K INT), compared to that in the outer area, which was 35 °C − 6.6 °C = 28.4 °C (marked as K EX).

**Figure 10:**
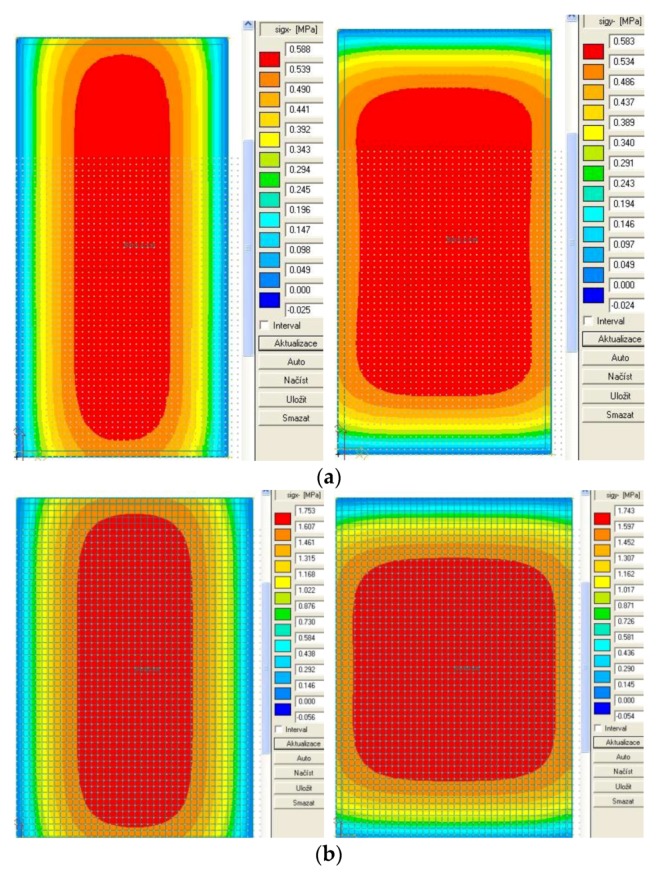
Stress distributions (σ_x_, σ_y_) in concrete slabs: (**a**) temperature gradient 0.3 °C per mm (K INT); and (**b**) temperature gradient 0.1 °C per mm (K EX).

Table 2 and Table 3 show an overview of calculated load stress for these two temperature gradients at the bottom of the slab. The stresses induced by the temperature gradient alone are shown in Figure 10a,b.

For the given conditions, the calculations of stress were made on the K EX and K INT structures. Regarding both structures, the length of slabs was 5.0 m and the width was 3.5 m. The stress at the bottom of the slab calculated while taking into account the temperature effect inside (K INT) was lower than when dealing with the pavement outside (K EX). This difference was based on lower temperature gradient and the fact that the structure (K INT) was designed for the central part of the tunnel. In contrast to the point of maximum load-induced stress, the temperature-induced maximum stress was in the center of the slab.

### 3.2. ANSYS Results

#### 3.2.1. Stress at Bottom Part of Concrete Slab

The calculation results of horizontal tensile stress at the bottom part of the slab joint under the dowels were only slightly influenced by the investigated misalignment positions of dowels, and they nearly always ranged 0.94–1.05 MPa for slabs of 300 mm thickness and 1.2–1.3 MPa for slabs of 250 mm thickness, which are low values (Table 4). In addition, with the typical increase by 50%, the tensile stress at the bottom slab (reinforced by dowels) did not exceed the value required for concrete pavement class I, i.e., 4.5 MPa.

#### 3.2.2. Stress around Dowel of Concrete Slab

**Figure 11:**
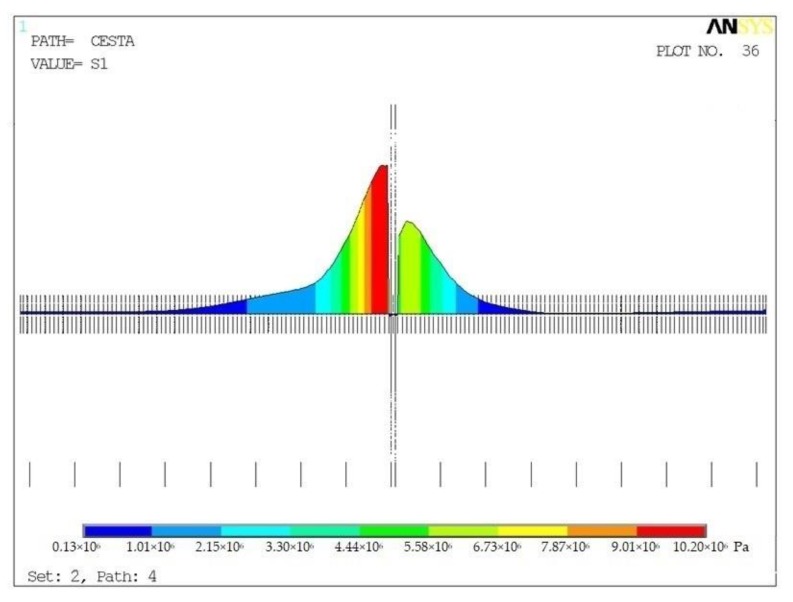
Main tensile stress in concrete for variant V12, Path 4.

The calculation helped to obtain the field of displacement, deformation, and stress for individual models (global and detailed), steps (ZS1 and ZS2) and variants. The FEM analysis determined tensile stress of the surrounding concrete for each dowel position (5.5–14 MPa) and initiated a potential risk of cracking (approximated tensile strength of concrete f_ct_ = 2.3 MPa) (Table 5). The course of stress in surrounding concrete (V1) is shown in Figure 11.

The lowest values of tensile stress were identified in Variants V10 and V11, when the dowel was tilted towards the load by the axle. The highest value of tensile stress was identified in Variant V14, when the dowel was tilted out of the load by the axle. Therefore, it was possible to see the highest sensitivity of tensile stress to the tilt of the dowel position.

The calculated extreme values of compressive stress had a similar trend (regarding the variants) to the one of tensile stress. In no variants did compressive stress (11–30 MPa) reach compressive strength of the material (f_ct_ = 32 MPa)—in accordance with the requirements of standard CSN EN 13877-1. Consequently, concrete cracking will not occur.

## 4. Conclusions

This paper presents main findings of FWD measurements on airport and road concrete pavements with used dowels in the Czech Republic and Slovakia. The results of the measurements show the low values of load transfer efficiency at transverse joints.

It is shown that software SCIA, ANSYS, etc. can be used for designing and assessment of concrete pavements. The causes were investigated with using the finite element method for modeling of stress on concrete pavement edges. The calculations were made in SCIA Nexis software. Modeling of stress conditions in the vicinity of in-built dowels was carried out by the FEM method in ANSYS software. The results were used for analyzing the importance of correct positions of dowels in concrete pavements and determining technological tolerances for their positions. In the Czech Republic, the permitted misalignment of dowel positions from the position designed in the documentation are defined in standard CSN 73 6123-1. Further research with laboratory tests (importance of position, diameter and quality of dowel coating) and measurements of deformations on real road pavements structures with the use of strain gauges will support theoretical results. Based on the performed experiments and modeling results, revision of allowed misalignment of the dowels could lead to updates of this standard.

The world research of dowels and experience with their application lead to using bigger diameter of dowels in relation to JPCP thickness and number of load applications. Therefore, the research results were applied in the revision of standard TP 098 Design of Road Concrete Pavements in Slovakia where 30 mm dowel diameter needs to be used for highways and allows designing and assessment of roads by the finite element method.

## Figures and Tables

**Table 1:** Material parameters and thickness of concrete pavement.

No.	Layer	Thickness (m)	Modulus E (MPa)	Poisson Ratio (-)
1	Concrete slab	0.24	37 500	0.20
2	Subbase (crushed aggregate)	0.30	600	0.25
3	Subgrade	2.50	120	0.33
-	Dowel	-	210 000	0.27
-	Dowel coating	-	200	0.40

**Table 2:** Calculated stress in concrete slab structure K EX.

Structure	Position of the Load	Stress (MPa) Load 100 kN	Stress (MPa) Load 115 kN	Stress (MPa) Temperature ^1^
K EX	center	0.781	0.881	1.753
	longitudinal edge	1.237	1.409	1.468
	transverse edge	1.272	1.446	1.404

^1^ Temperature gradient 0.3 °C per mm.

**Table 3:** Calculated stress in concrete slab structure K INT.

Structure	Position of the Load	Stress (MPa) Load 100 kN	Stress (MPa) Load 115 kN	Stress (MPa) Temperature ^1^
K INT	center	0.757	0.854	0.588
	longitudinal edge	1.232	1.337	0.482
	transverse edge	1.170	1.389	0.499

^1^ Temperature gradient 0.1 °C per mm.

**Table 4:** Results of stress at bottom part of concrete slab.

Variant	Stress σ_y_ (MPa) on Slab Bottom	Description
V1	1.386	Basic variant
V2	1.527	Missing adjoining dowel
V3	1.387	Vertical Translation
V4	1.387	Vertical Translation (upwards) 40 mm
V5	1.386	Vertical Translation (upwards) 60 mm
V6	1.397	Vertical Translation (downwards) 20 mm
V7	1.412	Vertical Translation (downwards) 40 mm
V8	1.425	Vertical Translation (downwards) 60 mm
V9	1.387	Vertical Tilt (away from the force) 20 mm
V10	1.390	Vertical Tilt (away from the force) 40 mm
V11	1.392	Vertical Tilt (away from the force) 60 mm
V12	1.385	Vertical Tilt (towards the force) 20 mm
V13	1.383	Vertical Tilt (towards the force) 40 mm
V14	1.380	Vertical Tilt (towards the force) 60 mm
V15	1.405	Longitudinal Translation (away from the force) 20 mm
V16	1.402	Longitudinal Translation (away from the force) 40 mm
V17	1.478	Longitudinal Translation (away from the force) 60 mm

**Table 5:** Results of stress around dowel.

Variant	Tensile Stress σ_y_ (MPa)	Compressive Stress σ_y_ (MPa)	Description
V1	10.2	26.7	Basic variant
V2	11.5	30.3	Missing adjoining dowel
V3	9.2	26.4	Vertical Translation
V4	9.4	27.0	Vertical Translation (upwards) 40 mm
V5	9.0	26.8	Vertical Translation (upwards) 60 mm
V6	10.9	26.5	Vertical Translation (downwards) 20 mm
V7	12.0	27.1	Vertical Translation (downwards) 40 mm
V8	12.4	25.1	Vertical Translation (downwards) 60 mm
V9	9.0	23.3	Vertical Tilt (away from the force) 20 mm
V10	5.7	17.6	Vertical Tilt (away from the force) 40 mm
V11	5.5	14.8	Vertical Tilt (away from the force) 60 mm
V12	10.2	23.4	Vertical Tilt (towards the force) 20 mm
V13	11.5	25.1	Vertical Tilt (towards the force) 40 mm
V14	14.2	26.6	Vertical Tilt (towards the force) 60 mm
V15	9.6	23.1	Longitudinal Translation (away from the force) 20 mm
V16	9.7	26.2	Longitudinal Translation (away from the force) 40 mm
V17	8.0	21.2	Longitudinal Translation (away from the force) 60 mm
